# A typology of useful evidence: approaches to increase the practical value of intervention research

**DOI:** 10.1186/s12874-020-00992-2

**Published:** 2020-05-28

**Authors:** Henna Hasson, Laura Leviton, Ulrica von Thiele Schwarz

**Affiliations:** 1 Procome research group, Department of Learning, Informatics, Management and Ethics, Medical Management Centre, Karolinska Institutet, SE 171 77 Stockholm, Sweden; 2 Unit for Implementation and Evaluation, Centre for Epidemiology and Community Medicine (CES), Stockholm County Council, SE 171 29 Stockholm, Sweden; 3 The Robert Wood Johnson Foundation, Princeton, USA; 4 School of Health, Care and Social Welfare, Mälardalen University, Mälardalen, Sweden

**Keywords:** Evidence-based interventions, Context, Implementation strategies, Core components, End users

## Abstract

**Background:**

Too often, studies of evidence-based interventions (EBIs) in preventive, community, and health care are not sufficiently useful to end users (typically practitioners, patients, policymakers, or other researchers). The ways in which intervention studies are conventionally conducted and reported mean that there is often a shortage of information when an EBI is used in practice.

The paper aims to invite the research community to consider ways to optimize not only the trustworthiness but also the research’s usefulness in intervention studies. This is done by proposing a typology that provides some approaches to useful EBIs for intervention researchers. The approaches originate from different research fields and are summarized to highlight their potential benefits from a usefulness perspective.

**Main message:**

The typology consists of research approaches to increase the usefulness of EBIs by improving the reporting of four features in intervention studies: (1) the *interventions* themselves, including core components and appropriate adaptations; (2) strategies to support–high-quality *implementation* of the interventions; (3) generalizations about the evidence in a variety of *contexts*; and (4) *outcomes* based on end users’ preferences and knowledge. The research approaches fall into three levels: *Description*, *Analysis,* and *Design.* The first level, *Description,* outlines what types of information about the intervention and its implementation, context, and outcomes can be helpful for end users. Research approaches under *analysis* offers alternative ways of analyzing data, increasing the precision of information provided to end users. Approaches summarized under *design* involve more radical changes and far-reaching implications for how research can provide more useful information. These approaches partly flip the order of efficacy and effectiveness, focusing not on whether an intervention works in highly controlled and optimal circumstances, but first and foremost whether an intervention can be implemented and lead to anticipated outcomes in everyday practice.

**Conclusions:**

The research community, as well as the end users of research, are invited to consider ways to optimize research’s usefulness as well as its trustworthiness. Many of the research approaches in the typology are not new, and their contributions to quality have been described for generations – but their contributions to useful knowledge need more attention.

## Background

Research on the effectiveness of health interventions (i.e., practices, treatments, programs, or policies) faces a critical dilemma: end users are frustrated and challenged. Practitioners, service organizations, policymakers and researchers alike often cannot use evidence-based interventions (EBIs), even when they are motivated to do so. The assumption is that EBIs are “plug and play,” but even the simplest EBIs often require careful deliberation in order to be adopted and effectively implemented. The necessary information to facilitate these goals – what we term “useful evidence” – is seldom offered in intervention research [1].^1^

An intervention’s outcomes are rarely caused by the intervention alone but rather by the joint forces of intervention plus context and implementation [2–4]. Thus, to provide useful information for practice, research needs to shed light on much more than the intervention. We believe that the challenges related to the use of EBIs arise from limited acknowledgement in the research community of four features: (1) descriptions of interventions, including the core components that are essential to achieve outcomes; (2) presentations of the strategies needed to implement the intervention; (3) understanding of the contexts in which the intervention is, or is not, effective; and (4) attention to the outcomes valued by end users. These features need attention when establishing the effectiveness of EBIs because the EBIs have no other justification than to be used.

Emerging methods to address these four features are scattered across multiple fields of research, which hinders learning across these fields. Furthermore, many of these advances target only a portion of the challenges we have described. For example, program evaluation recommends using logic models to describe the content of EBIs, but logic models give few insights about the contexts in which EBIs are likely to be effective. Quality improvement in medicine uses implementation strategies extensively and can rely heavily on the knowledge of both practitioners and patients, yet it often lacks theoretical or empirical underpinnings to understand effectiveness [5]. The current paper aims to invite the research community to consider ways to optimize not only the trustworthiness but also the research’s usefulness in intervention studies. This is done by proposing a typology that provides some approaches to useful EBIs for intervention researchers. This complements the substantive literature that focuses on improving the use of research evidence on the practitioner level by focusing on how usefulness can be improved in the production of research evidence.

### Challenges to established research pathways

The primary aim for conventional research is to test interventions in convincing ways: the statistical, clinical, or population health significance of outcomes. The established pathway requires interventions to first be carefully evaluated in efficacy studies, testing their ability to produce outcomes in a controlled environment and ruling out causal explanations other than the intervention itself. Thereafter, the interventions are supposed to be tested for generalizability in effectiveness studies, using more heterogeneous samples and contexts and often a broader range of outcomes, such as quality of life. After that, the interventions are assumed to be ready to be used by practitioners. Thus, whereas there are ample papers discussing research methodologies, they primarily focus on other aspects than the subsequent usefulness of the findings they produce.

However, this research process is far too slow: one estimate places the timeline for medical interventions – from primary study to uptake in guidelines – at 17 years [6]. After this is an equally bumpy road during which EBIs are to be implemented in clinical and community practice, resulting in a well-known variation between settings and patients [7]. Similar problems are seen in the uptake of evidence-based public health [8] and mental health interventions [9, 10] and in fields as diverse as education, criminal justice, and social welfare [11]. Some interventions are not adopted at all or take half a century to spread, such as Fairweather et al.’s model of a community-based “lodge” for people with serious and persistent mental illness [12]. Developed in 1963, a high-quality experiment revealed that lodge residents were less likely to need rehospitalization than those living individually, their employment was greater, and overall costs were lower than alternative interventions; however, the lodge model saw little uptake over the next decade. Half a century later, 13 states support the lodge model, yet it still serves only a small fraction of US residents with chronic mental illnesses [13]. These challenges in uptake have persisted despite methodological developments and the growth of research focusing on how practitioners' adoption of EBIs can be improved.

### The four interrelated features of useful research on evidence-based interventions

The ways in which intervention studies are conventionally conducted and reported mean that there is a shortage of information when an EBI is used in practice related to the four features thereof: the intervention itself, its implementation, its context, and the outcomes.

#### Description and specification of essential intervention elements

Limited descriptions of interventions hinder practitioners’ use of EBIs. Guidance or manuals of operation often do not clearly identify the core components of an EBI – also called its essential elements or central principles – that make the EBI effective [14, 15]. Ideally, these derive from a program theory or logic model, as simple prescribed activities do not cast light on the underlying mechanisms that link activities to outcomes. Without that deeper understanding, practitioners run the risk of replicating the outward trappings of interventions without the essential elements that make them work in context [5].

This information shortage frustrates not only practitioners who need more guidance to adopt an EBI [14, 16], but also researchers who want to replicate a study or categorize it in a systematic review [17]; this problem also applies to organizations that need to select the appropriate interventions for their situations [18], and policymakers who want to endorse interventions, fund them, or assist in implementing them [11].

#### Understanding of what is needed for high-quality implementation

Implementation of EBIs is complex in both clinical and community practice, but conventional research provides too little information necessary for practitioners to manage implementation [19, 20]. Local contexts often demand at least some departures from manuals of operations, but how to do so is seldom empirically tested or described. This is problematic, as end users need to determine how they can ensure that adaptations to an EBI improve effects [21, 22] or at least don’t impede them [23]. Unreported adaptations are also problematic for researchers because they are a barrier to drawing conclusions from systematic reviews: reviewers cannot gauge the intensity and duration of what was delivered, so they cannot explain variations in outcomes across studies.

Descriptions are also scarce concerning implementation strategies: the specific supports needed to assure high-quality implementation of an EBI and needed improvements in the setting, both organizational (e.g., leadership, climate for change) and individual (e.g., knowledge, skills, motivation). Implementation strategies can comprise a single activity or multiple ones – as is often the case – to strengthen the implementation process. When published studies are silent on the implementation strategies used and their impact, those considering the EBI do not know what these joint influences might be, and those adopting the EBI do not know what strategies they need to overcome barriers.

#### Understanding context in generalizing about EBIs

“Context” has become the catchphrase of the health field to explain why an EBI did or did not produce an effect. However, as researchers from a wide variety of fields have pointed out, context encompasses an enormous range of variables and unique interactions of patients, practitioners, the organizations in which they reside, the systems of which they are a part, and the era in which studies of EBIs are conducted [11]. While researchers understandably struggle with identifying the key features of context-influencing outcomes, practitioners need to act and have no choice but to make guesses about whether the EBI implemented in their own contexts will produce the same effects as in research studies.

Policymakers try to support practitioners by publishing practice guidelines, registries of tested models, and service payment requirements [11], but the underlying logic is shaky: just because five studies conclude that an EBI is effective does not imply that the EBI will also work in a sixth, different context. In fact, no table failures to replicate [5, 24, 25], as well as the inconsistent or even contradictory effect sizes often encountered in systematic reviews, support the shakiness of that logic. While some of these patterns are likely due to sampling error, they justify a more systematic inquiry into other forces at work.

An example of the hidden but powerful influence of context is the Nurse Family Partnership. Though this is deemed one of the best-documented EBIs in public health [26], a well-conducted trial in the United Kingdom failed to replicate the effects of the original US study [25]. Was this due to better existing services in the UK than in the US supporting new mothers? If so, then might there be a ceiling effect for maternal and child health? Or are UK practitioners stretched so thin – or are they so inured to mandated practices and policies – that even careful implementation of this EBI could not achieve its purpose? These are all plausible explanations, given other studies in the UK context [27], and they offer examples of how successful knowledge transfer across settings depends on information about contextual factors and their potential to interact with EBIs.

#### Understanding the outcomes that matter to end users

The starting point for intervention research is most often the researchers’ knowledge, rather than the experiences of a broader range of end users. This tendency threatens the relevance of EBIs and may also make the benefits of EBIs less convincing for end users if researchers fail to address outcomes that matter to end users, or fail to address how the EBI stands in relation to the programs currently in use.

By addressing questions about the relevance, applicability, and usefulness of EBIs upstream – that is, during the development and testing of interventions – many challenges of using EBIs in practice will be circumvented. Furthermore, involving end users (patients, professionals, and policy makers) in research ensures that their knowledge and buy-in are incorporated early.

## A typology of useful evidence: overview

The typology provides a classification system for intervention research approaches based on how they contribute to the usefulness of EBIs. The typology covers research approaches on three levels, as seen in the columns of Table 1; *Description*, *Analysis,* and *Design* reflect incremental steps in single studies or a program of research on an EBI. The three levels aim to improve usefulness in different ways. *Description* outlines what types of information about the intervention and its implementation, context, and outcomes can be helpful for end users. Research approaches under *analysis* offer alternative ways of analyzing data, increasing the precision of information provided to end users. Conventional randomized controlled trials (RCT) are also analytic, but the typology’s approaches probe further than efficacy and effectiveness in determining for whom, when, and why an EBI works. Approaches summarized under *design* involve more radical changes and far-reaching implications for how research can provide more useful information. Approaches proposed under *design* partly flip the order of efficacy and effectiveness, focusing not on whether an intervention works in highly controlled and optimal circumstances, but first and foremost whether an intervention can be implemented and lead to anticipated outcomes in everyday practice (e.g., [28]).

**Table 1:** A Suggested Typology of Useful Evidence

	DESCRIPTION	ANALYSIS	DESIGN
Intervention	Describe the theory behind the EBI and the core components and program logic (intended and actual). Determine appropriate adaptations to the context.	Analyze the impact of core components and test the pathways outlined in the program logic.	Design to test variation in content and dose.
Implementation strategies	Describe the type and function of the implementation strategies (planned and actual).	Analyze the impact of implementation strategies on outcomes.	Experiment with implementation strategies and tailor them to the context.
Context	Describe the salient features of the context and why they are important to the outcomes (moderators).	Analyze how the contextual factors moderate outcomes (barriers and facilitators).	Design to test matching interventions to known moderators.
			Design to test the intervention in clinical practice and in community settings.
Outcome	Measure and report (1) all outcomes outlined in the program logic model, (2) the implementation outcomes, and (3) the value that end users place on the outcomes. Monitor unintended consequences.	Analyze how the intervention, implementation strategies, and context interact to produce outcomes.	Study the trends by using time-series data and integrated data systems, which can be used to improve care at the single-patient and group/system levels.

For each of the three levels, the typology considers the four features (the rows of Table 1): (1) intervention, (2) implementation strategies, (3) context, and (4) outcomes. These four features are derived from change management and implementation science models outlining how outcomes are affected not only with the content of change (i.e. the intervention), but also the process (i.e., implementation) and the context in which the change takes place (e.g., [29]). In the typology, *intervention* refers to the content of the EBI, its delivery format and intensity, appropriate adaptations, and the mechanisms linking the EBI to outcomes. *Implementation strategies* refer to the supporting activities performed to integrate the EBI into clinical or community practice [30]. *Context* refers to everything that can influence the effectiveness of an EBI that is not part of the intervention or implementation strategies [31]. Context refers to both inner organizational (e.g., structural, cultural) and outer (e.g., broader economic, political, and social) context [32], as well as to the practitioners and patients or communities receiving or using an EBI. The four features of the typology at the three levels are described below with examples of research methodologies as well as practical applications from the literature. The examples are illustrative, not systematic, aimed to provide insights of how the approaches have been applied from the fields of medicine, psychotherapy, nursing, behavioral health, public health, community-based prevention program evaluation, and implementation science.

### Level 1: description

The primary aim at this level is to provide end users with the information they need to translate research findings into practice [33]. Improved descriptions will require fairly small and relatively inexpensive inquiries. These can be mixed-method supplements to conventional efficacy and effectiveness research, e.g., identifying the core components of the Transitional Care Model [34]. They can also be freestanding supplements to inform a body of research on an EBI, like focus groups on obstetricians’ reluctance to use corticosteroids [35]. Whether qualitative or quantitative, process evaluation methods are descriptive in that they gather information on implementation and context concurrent with evaluation of outcomes [36]. Process evaluation only rises above the descriptive level when it is analyzed for its association with outcomes, or when it is deliberately manipulated through design (see below). Prospective descriptions are preferable, although post-intervention descriptions can also be valuable in providing comprehensive information. Existing guidelines for reporting interventions such as SQUIRE guidelines can be excellent tools to offer guidance in how to describe the study so that the end users get useful information [37].

#### The intervention: description of the core components and program logic

Only about one third of published EBIs in medical care are adequately described [38, 39], despite influential guidelines for reporting interventions [37, 40–44]. For an EBI to be useful, researchers need to clarify the theory underpinning the EBI and outline the program logic explicitly [11]. As SQUIRE guidelines suggest, the description of the intervention should be in sufficient detail so that others can reproduce it [37]. Logic models are a typical feature of program evaluation reports [45, 46] and is highly valued by end users in U.S. non-profit organizations [47]. Study protocols are another outlet for such information: thorough intervention descriptions help end users make sense of findings, derive a consensus on meaning, and convey it to outsiders [48].

Importantly, descriptions of core components should also include not only the plan, but also information about actual intervention content as it was implemented, including departures from the plan and the reasons behind them. This means documenting changes to the content, format, timing, and delivery [49]; for example, Hasson et al. first described planned core components and program logic of a preventive intervention for frail older people in a study protocol [50], and empirically evaluated the actual implementation and fidelity in a later study [51]. Using a combination of data sources revealed that although the fidelity was high, adaptations to the intervention were nevertheless made and new components added by the professionals providing the services to further improve outcomes. Without the observations of the actual intervention delivery and these added components, the study could have drawn false conclusions about the effectiveness of the intervention.

Information about how activities in the intervention are carried out is also crucial. The end user needs to know how each intervention component is to be performed in practice. An example of such detailed guidance is Project ALERT [52], an EBI that prevents substance abuse in grades 7 and 8 by addressing teens’ pro-drug mindset. A detailed logic model links theory to activities and to the desired outcomes in terms of changes in students’ attitudes, beliefs, and behaviors. Lesson plans, demonstration videos, and the principles behind each activity are presented in detail. The user is guided at each step to understand what fidelity to the model is, and which departures will compromise outcomes. These materials were developed over a period of time and underwent laborious testing to understand the sequence of activities and their purpose in context; this labor-intensive work was valuable to the scores of practitioners who are confident they can use these materials effectively.

#### Describe implementation strategies

The activities that support the use of a certain EBI – i.e., the implementation strategies – may have an impact on how the intervention is used and the outcomes achieved [30]. Thus, activities such as training, technical assistance, and reminders need to be reported carefully so that end users know what supporting activities might be needed [53, 54]. We suggest that the descriptions include both the implementation strategies planned and those actually used (as plans can change), and that researchers report them as carefully as the content of the intervention [49]. This is seldom the case: implementation strategies are usually not described in any detail in scientific journals [55, 56].

Implementation science literature also suggests standardizing the descriptions of implementation strategies in order to be certain that various studies use the same strategies in the same ways, using the same label [30]. Standardized descriptions facilitate the usefulness of research findings by painting a more complete picture of studies, enabling comparisons across studies, guiding end users for implementation, and improving accountability [30, 57, 58]. Examples of standardization include Powell et al. [58], who compiled 68 discrete implementation strategies into six categories: (a) plan, (b) educate, (c) finance, (d) restructure, (e) manage quality, and (f) attend to policy context. Another way to standardize descriptions of implementation strategies was provided by Michie et al. [59], who proposed a systematic and detailed way of describing implementation strategies by defining their function. For instance, they propose that staff training needs to be further specified according to its active pedagogic ingredients – such as role play, modeling, and feedback – and intended function, such as increased capability or improved motivation. In program evaluation, such functions are termed short or intermediate outcomes, while in medicine they are mechanisms to achieve effects. These mechanisms can operate at various levels: intrapersonal (e.g., learning), interpersonal (e.g., sharing), organizational (e.g., leading), community (e.g., restructuring), and macro-policy (e.g., guiding) [60].

#### Description of context

A careful context description helps to clarify the generalizability of the results [11] and means that decision-makers and professionals who intend to use an intervention can assess its feasibility for their settings [18, 54, 61]. Decision-makers and professionals want to understand under what circumstances the EBI has been shown to be effective, how those circumstances differ from their own situation, and what contextual factors can influence its implementation and/or outcomes [62]. Yet context is often discussed briefly as a limitation to generalizability, without further probing about *why* generalizability might be limited to this particular context.

As with implementation strategies, guidance is available to define and describe context in order to maximize value for end users (e.g. [32, 63]). One of the most comprehensive models is the consolidated framework for implementation research (CFIR) [32], which categorizes context as outer and inner settings of an organization [64]. The outer setting refers to the political, economic, and social context, such as networking with external organizations, as well as external policies and regulations to promote certain implementations [64, 65]. The inner setting refers to the structural characteristics of an organization, such as its size and location, as well as modifiable factors, such as the organizational culture and contextual climate in which the implementation takes place; thus, this framework consists of a list of factors in a context that might be relevant to report in an intervention study. The categories described in the CFIR can provide guidance on what aspects of context might be relevant to observe and describe in intervention research.

Contextual factors offer a challenge, however, because lists of such factors are expanding, but measurement of every factor in every study is not possible. Neither resources nor statistical methods make comprehensive measurement desirable, and even if all factors were measured, some unknown factors might still be missed. Rather, we propose that such lists can function as guidance in selecting the contextual factors that are most relevant in specific settings. The Pareto principle (the so-called “80–20 rule”) justifies decisions to limit the number of factors to be measured. It states that the large majority of effects have a relatively small number of causes. To apply the Pareto principle, researchers and end users must make informed judgements about factors’ plausible importance and the frequency with which they are encountered. For example, the failure to replicate the Nurse Family Partnership in the UK gave rise to several plausible explanations about context (these might be testable at the levels of analysis and design) [25].

Judgements can be informed by consulting the literature on the intervention, surveys of end users, and studying implementation with qualitative methods such as ethnographical approaches. End users become an important resource for this purpose because collectively, they have experienced more settings and contextual factors than have researchers. The Transitional Care Model offers an example, because surveys of practitioners helped to identify the frequent and important barriers to implementation, which users could then address strategically [66]. Any criteria for selecting contextual factors are imperfect, but prioritizing usefulness makes the choices more systematic.

#### Description of outcomes

There are three main ways in which description of outcomes can improve the usefulness of EBIs: (1) by reporting on outcomes that matter to end users (regardless if the outcomes are intentional or not), (2) providing information about the implementation outcomes, and (3) reporting all the outcomes (both proximal and distal) outlined in the program logic (regardless of whether or not they are significant).

To fulfill the first condition, researchers need to collaborate with end users on outcomes, an increasingly common practice on online platforms, forums, and other media. Advocates for people living with chronic conditions are more involved in the choice of outcomes to be studied [67], and community participants are invited to guide choices in public health research [68], just as Patient-Reported Outcome Measures (PROMs) are believed to be more meaningful to end users [69].

Engaging end users may be one way to identify both intended and unintended outcomes, as well as wanted and unwanted ones. This information can be further accentuated by considering outcomes that matter for different stakeholders: patients, professionals, and organizations delivering the EBIs, as well as system representatives (e.g., policymakers and citizens). Measurement of different types of outcomes is crucial because outcomes can be contradictory to different stakeholders’ interests. For instance, comprehensive treatment may be clinically effective (and thus be valued by patients and professionals), but may prolong waiting times or increase costs, which is detrimental to organizations, existing systems, and patients not already receiving treatment.

New ways in which researchers can engage with end users have also been developed. Von Thiele Schwarz et al. developed a process labelled COP (Co-created Program Logic) as a way to identify the outcomes valued by multiple stakeholders in a health system [70]. The aim was threefold: to inform evaluation by identifying outcomes relevant to stakeholders, promote a shared understanding of outcomes across the stakeholder groups, and build acceptance for the result of the evaluation. COP is done in a half-day workshop, to which representatives of all relevant stakeholders are invited. Stakeholders work together to identify outcomes that matter and discuss how outcomes are related to each other and the core components of the intervention. The end product is a co-created program logic that stakeholders have bought into, informing researchers what outcomes they should evaluate.

Implementation outcomes are another factor important to end users, crucial to understand what reactions the EBI evoked in professionals and patients and how the intervention’s core components were expressed in reality (e.g., how the intervention was delivered and received) [45]. Proctor et al. proposed a total of eight implementation outcomes that give early information about how the intervention is perceived and used [71].

Last, it is also crucial for end users to receive information about both proximal and distal outcomes outlined in the program logic, regardless if they improved significantly or not. These outcomes may include acceptance of and exposure to the intervention, behavior or lifestyle changes, clinical improvements (e.g., patient symptoms), services (e.g., costs or number of patients treated), systems improvements (e.g., access or reach of services), improved patient health, or population-level health indicators [71, 72].

### Level 2: analysis

At this level, intervention research can become more useful by providing more concrete knowledge about the EBI and its implementation, context, and outcomes. Descriptions can provide clues about how an EBI works, but analysis establishes whether, how, and why it works; this requires a thoughtful application of both qualitative and quantitative data. For instance, interview data can provide insights into the intervention users’ experiences about the mechanisms for change, which is essential for developing theory. Concurrently, statistical analysis can provide tests of theoretical propositions and quantify the relationships involved [73, 74].

#### The intervention: analyses of core components and program logic

To further investigate how an EBI works, one can analyze which core components are necessary to achieve outcomes, and how well the logic chain of proposed mechanisms holds up [75, 76]. Such approaches give insights into why an EBI leads to certain outcomes. For example, Querstret et al. [77] developed an Internet-based, instructor-led mindfulness intervention on recovery from stress. The intervention consisted of multiple core components, but only one, “acting with awareness,” explained the outcomes. This finding led the investigators to revise their view of what was essential for mindfulness interventions; thus it not only informed their underlying theory of mindfulness, but also meant that the EBI become simpler, more accurate, and more cost-effective. This is a big step towards more useful evidence. With efficiency in mind, Collins et al. have developed a staged process of dismantling and testing prevention EBIs, starting with the logic model and systematically eliminating core components to arrive at an optimized version [78]. This kind of investigation also helps identify which components need be implemented with fidelity, and what can be adapted.

Probing and sharpening the underlying theory has other practical advantages. A well-tested theory builds confidence about why apparently different interventions produce similar effects and by extension helps identify components that are common between different interventions [79]. AIDS prevention offers an example: engaging people at risk often takes place in venues of importance to them and relies on locally relevant content; therefore, benefits and risks need to be conveyed in people’s own terms, and support needs to be tailored to overcome barriers like addiction or partner violence [15].

#### Analyses of implementation strategies

As with core components, one can analyze how various implementation strategies affect outcomes and how they may interact with the EBI’s core components and context. This type of information suggests to end users how the intervention’s components can be applied in different environments. One can, for example, investigate whether staff skills training strengthens the impact of an intervention. Implementation studies are nothing new, but are often done separately instead of in parallel or integrated with outcome evaluations, potentially missing an opportunity to learn about these linkages [80].

Boyd et al.’s [81] study of measurement-based health care (MBC) illustrates the advantages of integrating outcome and implementation studies. They conducted preliminary examinations of the association between implementation strategies and self-reported fidelity in MBC. Although quality management, environmental restructuring, communication, education, and planning were more common implementation strategies than financing, the latter was the only strategy that was associated with improved fidelity. This sort of finding is very helpful to end users.

#### Analyses of contextual factors

End users are often concerned with understanding the circumstances under which an intervention works best [73, 82]. Common quantitative approaches include analysis of subgroups (the intervention works best for subgroups of a larger study sample) and of moderators [82, 83]. These analyses can make research findings more useful by going beyond an overall group mean value to a more specific estimate for sub-groups and situations. Identification of moderators can illuminate how widespread an intervention’s effects are, how robust they are under different conditions, and whether the effects are similar across different kinds of patients [83]. This may, for example, involve investigating whether smokers react differently to a treatment than non-smokers do [83]. Analysis of contextual influences does not have to be limited to patient characteristics. Organizational and community factors, such as leadership, group climate for change, and participants’ readiness for the intervention, can affect outcomes. Thus, moderators may be found on both the individual and the unit, organizational, or community levels, calling for multilevel moderator models [84]. Nevertheless, given the multitude of possible influencing contextual factors, each study will likely focus on a subset of factors.

Many studies of moderators have limited practical value because they investigated one moderator at a time, were limited to a single study and outcome, or had small statistical effects [75]. Combinations of moderators might better explain the results [82]. Recent statistical developments have been promising for providing better information on context for clinical decision making. They allow a comparison of individual moderator effect sizes. Also, diverse moderators can also be analyzed in composite to explain outcomes [82].

Earlier, we alluded to the problem that many studies on effectiveness take place under ideal conditions, such that the results would not be generalizable to lower capacity settings and practitioners. Shadish and colleagues [85] identified a way to address this problem using meta-analysis. For many years, psychotherapists objected to the conclusion from meta-analysis that psychotherapy is effective because so many effectiveness studies were conducted by highly motivated, newly trained clinicians under optimal, supervised conditions. When Shadish and colleagues [85] re-examined studies on the effects of psychotherapy across a range of representative clinical contexts, they concluded that its effects were robust across real-world conditions.

#### Outcomes: the combined effects of study features

Instead of testing whether individual study features affect outcomes separately, one can test whole configurations of interventions, implementation, and contexts and how these interact to produce an outcome. These approaches build on the assumption that few interventions work for everyone and that some interventions work in some contexts but not others [86, 87].

Realist evaluation is an example of how whole configurations can be tested. The starting point is a hypothetical program logic that outlines what works for whom and when. For instance, the logic for an intervention to increase parental involvement in children’s school work might specify that 1) parents who lack confidence in their own ability (context) 2) need to feel included and welcomed by school staff (mechanism) 3) to come to the meetings at the school (outcomes). This program logic can be empirically tested, for example, through interviews to see which mechanisms generate the outcomes in that context [88, 89].

Mediated-moderation analysis is another way to test, statistically, what works for whom and when. Bond and colleagues [90] provided an occupational health example. Their intervention on work reorganization was aimed at improving mental health and absence rates in a call center. The logic pathway tested if employees’ psychological flexibility would moderate the intervention’s effects and whether changes in outcomes were mediated by changes in job control (which the intervention was aimed at improving). The model found support in the statistical analysis: the intervention enhanced perceptions of job control and subsequently the wellbeing outcomes, especially for those who had greater psychological flexibility. Thus, moderated mediation models provide specificity with which to draw conclusions about the causal direction of effects, by analyzing the influences of contextual and implementation factors, rather than controlling them.

### Level 3: design

The design level can potentially increase the usefulness of research findings more than the description and analysis levels can because usefulness considerations are incorporated into planning of the study design. Various disciplines, motivated by the challenges of using evidence, have developed diverse approaches to do so. We can see two main categories: those that aim to increase, or else decrease, researchers’ control over factors influencing the outcomes. Many fields of clinical science suggest designs to experimentally test different versions of EBIs, which are examples of the first category [91] (see below). Others advocate more natural experiments in which researchers do not exercise control to test different versions. Instead, they carefully document the naturally occurring practice variations [92, 93]. Both of these approaches are valuable, as seen in the examples below.

#### Intervention – controlled experimentation

One way to better understand how and why an intervention works is to actively vary the intervention components and doses. Participants may be randomized into different versions or exposed to varying levels of intervention intensity and duration. Two examples of controlled experiments with intervention dose will be given. The first comes from medicine: Gravenstein et al. [94] compared elderly nursing home residents receiving standard doses and high doses of influenza vaccine to investigate which dose was more effective in reducing the risk of respiratory-related hospital admissions. The rationale was that immune responses to influenza vaccines decline with age, reducing their clinical effectiveness. The higher dose was found to be more effective for this population. Wilcox et al. [95] studied an educational EBI to maintain physical activity in people over 50. They also tested the dose and found that reducing the number of group sessions by about a third made no difference to the outcomes at 6 months. Because time and resources are often key constraints, this finding made it possible for a wider variety of nonprofit organizations to implement the EBI. Although the study was not an RCT, the findings were practically significant because having fewer sessions meant that working people could more easily attend them.

Controlled experiments can also be used to unpack interventions consisting of several components. Researchers generally study the effects of the components together as a package, but as a result, the impacts of individual components and their relative importance remain unknown. This type of component analysis has been suggested as one of the most important aspects in developing evaluations of treatment effectiveness, such as psychotherapies, and for introducing the interventions into clinical practice [91]. Component analysis does not necessarily require large samples. For example, Villatte et al. [96] studied 15 individuals seeking mental health treatment. They were randomized to one of two modules of acceptance and commitment therapy (ACT): either one focusing on acceptance and cognitive defusion (seeing thoughts as thoughts, not as realities) (ACT OPEN) or one focusing on value-based activation (i.e., spending time on activities one values) (ACT ENGAGED). Both of the modules led to fewer psychiatric symptoms and improved quality of life, as compared to before the treatment, but importantly, the proposed mechanisms were shown to differ between the two groups. ACT OPEN improved ratings of acceptance and cognitive defusion, while ACT ENGAGED improved value-based activation. As this example illustrates, it is possible to contribute information that is highly useful for practice by explicating and testing a theory-based mechanism, using repeated data, even without large datasets.

From a practice perspective, each component of an intervention adds to the complexity of using the intervention. Without guiding information about different components, interventions as a whole may risk not being implemented at all, or the implementation can become unnecessarily complicated or lengthy.

Another area of research emphasizing experimental testing of different versions of interventions is culturally adapted interventions [97–101]. The starting point is that most interventions are designed for, and tested with, homogeneous majority populations and then expected to be used for other populations for which the interventions have not been evaluated. This line of research has suggested that interventions should be carefully adapted to fit to the needs of a specific minority group and experimentally tested alongside the original intervention to compare their outcomes. Cultural adaptation requires consideration of the end users’ needs and values, which in turn requires close collaboration between the end users and researchers. In this way, an EBI tested on a majority population can be compared with one that clearly takes cultural and practical circumstances into account. An important task for researchers in this research stream is to empirically investigate the acceptable boundaries for core component adherence and flexibility [102].

#### Intervention – natural experiments

The second stream of approaches to designing studies with increased usefulness has suggested more natural experiments. In this approach, the intervention is allowed to vary both in content and dose, just as it does when used by professionals in real-world practice settings. The justification is that practitioners need to understand how the intervention is used under real-world conditions and the outcomes obtained with different versions of the EBI.

This does not necessarily mean that no control is imposed; instead, the degree of control can vary. For instance, in the step-wedge design, people or clusters (such as clinics) are randomized to begin participation at different time points [103]. Some have suggested that the step-wedge is appropriate to study interventions that evolve over time, provided that there is no requirement to “freeze” the intervention [104]. With this design, an intervention evolves over a series of tests, and outcomes are analyzed with multiple interrupted time-series [105]. For example, Bailet et al. [106, 107] conducted a three-year step-wedge intervention design to teach emergent literacy skills to preschoolers who were considered at risk of reading failure. During the first year, they compared randomly allocated children to spring and autumn groups (and a control condition), which enabled analyses of effect maintenance between the groups. New students were added during each successive year, and changes were made to both the measured outcomes and the lesson content. These successive changes allowed the researchers to improve the content, evaluate a variety of outcomes, and measure both the duration of effects and when in the process they might be expected.

Others have started with natural variations in clinicians' daily practice. For instance, Galovski et al. [108] tested, in a randomized, controlled semi-crossover design, a flexible approach to a cognitive processing therapy (CPT) intervention. They allowed the professionals to use their clinical experience to determine the number of treatments (between 4 and 18) based on the patients’ recovery status, defined as the individual participants’ accomplishment of an *a priori* defined, specific end-state criteria. This was compared against a standard 12-session protocol. The majority of the participants reached the end-state criteria prior to the 12th session and also maintained their treatment gains at the follow-up measurement. This is an example of an intervention study providing practical applicable research findings while also being conducted using a strong, high-quality study design.

Additional designs that embrace natural variation include approaches comparing new interventions to interventions that are already being used in practice. These approaches are common within the fields of pragmatic trials and comparative effectiveness research [109, 110]. One justification for these approaches is that given all of the resources needed to put new interventions into place, it is not enough for a new intervention to be effective; rather, it must be far *more effective* than the alternatives already in use. From this line of reasoning, it can also be argued that a new intervention should be compared with the *best* alternative that is currently used in clinical practice.

#### Experiment with and tailor implementation strategies

In line with the suggestions for experimenting with different intervention content and doses, implementation strategies can also be tested experimentally. For example, groups can be randomized to receive one of two implementation strategies, e.g., reminders and performance feedback to study the degree of implementation and distal outcomes [111]. Alternatively, a Cochrane Review recently recommended [112] a more sophisticated version in which the implementation strategies are tailored, chosen based on local needs, obstacles, and possibilities for changes [112]. This may involve analyzing the level of staff competence, the patients’ expectations, or the organization’s capacities and matching implementation strategies based on them [112, 113]. This recommendation to use tailored implementation is based on implementation research showing that implementation strategies are equally (in) effective if not based on the needs and circumstances in the current context [112].

It may not be necessary for researchers to independently decide upon which implementation strategies to use. Instead, that decision may be left to the organizations involved or be determined in collaboration between researchers and the practitioners. For example, Sinnema et al. [114] evaluated the impact of tailored implementation on primary care physicians’ diagnosis and treatment of anxiety or depression. They used a clustered randomized controlled design with 46 GPs from 23 units (12 intervention, 11 control) and 444 patients. In the standardized implementation group, GPs received a 1-day training session on clinical guidelines for anxiety and depression as well as continuous feedback on their performance. In the tailored group, GPs received the same training and feedback, together with support that was tailored to their specific personal barriers in using the guidelines. The barriers were identified in pre-intervention interviews with the GPs and classified by theme, such as knowledge and skills, time constraints, patients’ attitudes, collaboration with mental health professionals, and availability of treatment. Better implementation outcomes were observed for the tailored – as compared to the standardized – implementation group. In this example, the premises and needs of the end users were incorporated into the research design, resulting in implementation strategies that improved outcomes.

#### Design to test variation in context

Two approaches for testing contextual influences can be identified in the literature: matching the interventions to known moderators and designing interventions directly in clinical practice.

##### Matching interventions to known moderators

Known moderators of certain intervention components can be used when developing an intervention and when evaluating the components’ effects [74, 115]. For decades, psychotherapy research has used evaluation designs in which patients with certain characteristics receive certain treatments. For example, Öst et al. [116] studied the impact of a cognitive behavior therapy (CBT) intervention for claustrophobia, taking into consideration the patients’ response patterns to being in tight spaces. The patients went into a small space, and their behavior, heart rate, and experience of anxiety were measured. Patients who had strong avoidance behavior but a small increase in pulse were categorized as behaviorally reactive, while those who had a strong pulse increase but little avoidance behavior were deemed to be physiologically reactive. The patients from these two groups were then randomized into treatments with either exposure or applied relaxation. The hypothesis was that exposure would have better results than relaxation for the behaviorally reactive, whereas relaxation would be better than exposure for the physiologically reactive. The results were fully in line with the hypotheses, showing that matching the treatment to the response pattern improved the outcomes. Such studies provide actionable information for end users, by providing trustworthy guidance for how they can tailor interventions to patients.

Recent developments in both psychotherapy and medicine have taken the matching of moderators to interventions further still, in so-called individualized treatment or personalized medicine [75, 117]. With these approaches, the aim is to tailor interventions to subgroups or even individuals, based on their unique situations. In the long term, this could broaden and deepen the information on interventions and provide tools with which to adapt them to various patient segments and individuals [83].

One objection to individualized approaches is that the study may inject bias into data collection and analysis. As with all tests of research hypotheses, blinding the data collection and analysis to the condition will reassure end users. An example is an RCT of multifaceted quality improvements to surfactant therapy in preterm infants, which achieved a far larger effect size for practice changes compared to other studies at the time [118]. Given the potential benefits from such approaches, however, blinding should not be a precondition for doing a study.

##### Test interventions in routine clinical practice

The usefulness of research findings can also be improved by designing intervention studies directly in clinical practice, making the context an integrated part of the study. This approach involves adopting some aspects of pragmatic trials [119], in which the intervention is tested in a clinical context similar to where it is to be used, rather than a context designed or controlled by the researchers. Representative participants and settings are prioritized. For example, all patients seeking the service are included, no strict exclusion criteria are applied, and no special recruitment methods are used. To fully study an intervention in its clinical context would also imply that the intervention is implemented within the scope of the organization’s existing resources. No extra measures that are not in place in normal clinical practice are taken to support the intervention’s use, in order to secure high representativeness.

Price et al. [120] provides an example of a pragmatic trial focusing on maximizing external validity. They studied a heterogeneous real-world population to explore a question that they considered would not be possible to be answered in more tightly controlled randomized controlled trials – namely, the effectiveness of proven asthma therapies for regular primary care patients, including those who smoke and those with coexisting conditions, poor adherence, and poor inhaler technique. In most prior trials, as much as 95% of asthma patients had been excluded, including smokers, despite smokers making up one fourth of the patient population. They conducted two pragmatic trials to evaluate the effectiveness of different asthma treatments, which included broad groups of patients (ages 12–80) with asthma. The patients were randomly assigned to one of three asthma treatments for 2 years of open-label therapy, under the care of their usual physician. Interestingly, little difference in real-world effectiveness was found between the treatments, which challenged the guidelines for asthma treatment. Thus, caution should be applied in extrapolating results from randomized clinical trials to the broad population of patients with asthma. The authors suggested that the clinical decision-making can be best guided by viewing the results of conventional randomized controlled trials, in conjunction with the results of pragmatic trials.

A last example of how the usefulness of evidence can be improved by design is to ensure that usefulness is already a criterion in an intervention’s development. This means that factors that may make the intervention challenging to implement and use must already be addressed in the intervention’s development. Lyon and Koerner [121] suggest that intervention developers apply user-centered design principles for this, including 1) identifying the end users and their needs up front, 2) using prototyping and rapid iterations, 3) simplifying existing intervention components, and 4) exploiting the constraints inherent in typical use contexts. An assumption behind these approaches is that a simpler intervention that is seemingly less effective may be preferable to a more complex one that never stands a chance of being used in practice anyway. Engaging end users in the intervention development is thus a way to fix some of the challenges encountered upstream in the research-to-practice pathway.

#### Outcomes – measuring temporal sequences

Measuring outcomes at multiple points has many advantages for the usefulness of research findings. Multiple measurement before and after an intervention has long been known to control for a variety of alternative explanations for results. Having several measurement points potentially decreases the risk of drawing incorrect conclusions about the intervention’s effects due to temporary circumstances in connection with the measurement occasion [122]. It also allows the change process, or trends, to be studied in more detail [115]. This can illuminate whether the change occurs at different time points for different participants. Some might be late bloomers, while others may first improve and then regress [115, 123]. Walraven et al. [124] provide an example of how time-series analysis was used retrospectively to study naturally occurring changes when randomization was not an option. They revealed how different changes in policies (e.g., guidelines) changed physicians’ laboratory orders during a period covering more than 6 years. They had data on counts over time of the most common laboratory tests in the region and were able to pinpoint how the policies reduced the volumes of several tests during those years.

Furthermore, using prospective data allows the development of individuals or clusters of individuals to be followed up over time, i.e., individual trajectories. Similarities in baseline characteristics for these individuals can provide information about important moderators, such as by indicating which groups are more likely to benefit from the intervention. Leon et al. [125] used a group-based trajectory model (latent class growth analysis, LCGA) to investigate possible diversity in the change courses of psychiatric acuity among children during hospitalization. The acuity of psychiatric illness was measured every day for each patient, and the LCGA allowed analysis of the probability that each person belonged to a particular trajectory, based on the similarities and differences in their scores. Only one of the identified seven patterns was linear (i.e., linear improvements from baseline), while four were quadratic (i.e., non-linear; the so-called honeymoon effect of getting initially better but quickly worse again) and two were not associated with a significant change at all. This study illustrates how rigorous statistical evaluation using continuous data on individuals can reveal sets of patterned response trajectories.

Another type of design that benefits from continuous data is single-subject designs, in which an individual is his/her own control by using multiple measurements over time. This implies that one or a few participants are followed individually, rather than studying means of groups. Single-subject designs can also be used to change the experimental condition with a controlled condition, such as by introducing an EBI and then withdrawing it (e.g., ABAB designs). Drawing single-subject designs even further, recent developments in digital decision support systems suggest using continuous measurement of individuals’ development along with a comparison to the expected outcomes. This can provide opportunities to change the intervention content or exposure levels if the expected results are not obtained [126].

## Discussion

Intervention research is expected to provide both valid conclusions and useful findings [127]. The current paper invites the research community, as well as the end users of research, to consider ways to optimize both usefulness and quality.

The proposed typology outlines three levels where researchers can increase the usefulness of their studies (by describing, analyzing, and designing); and by clarifying four features that can be improved: intervention content, implementation strategies, context, and outcomes. Yet, the three levels are not mutually exclusive. Rather, one needs to describe to be able to analyze, and if a new design is to serve any purpose, one needs to both describe and analyze when using new designs. The examples provided of research approaches are in no way complete, and we recommend scholars to continue develop our understanding of the usefulness of different research methods, including conducting systematic literature studies. Yet, the examples illustrate approaches that may be applicable to a variety of fields and topics. Our aspiration is that all intervention researchers, regardless of study type, setting, or intervention, should be able to use at least some of these approaches.

The research approaches presented in the typology may provide ways to balance internal and external validity in a given study or research program, a challenge that goes to the very heart of what constitutes usefulness and quality. At the *description* level, usefulness comes from adding information about the intervention content, context, and implementation strategies as well as from more careful selection and reporting of outcomes, in collaboration with end users. At the *analysis* level, the focus is on both understanding whether the program works (internal validity) and also for whom it works and how, contributing more to external validity than most research on EBIs does. The priority given to external validity is greatest at the *design* level, based on the argument that conducting studies that are not manifestly useful in practice is meaningless. Some of the proposed approaches (e.g., pragmatic trials) might imply a risk of focusing too much on real-world practice and sacrificing internal validity to achieve generalizability. Proponents of conventional trials challenge such approaches because they tend to pose problems for causal inference [128]. Yet, any single study will have both advantages and disadvantages for such inferences, which is why scientists rely on a body of evidence rather than single studies.

Given the multitude of factors influencing the outcome of each intervention study, we do not propose that every single study address all aspects raised in this paper. Instead, the aspiration is that the usefulness of intervention studies will gradually increase through the accumulation of studies contributing to a more and more granular understanding of the influence of intervention, implementation and context on outcomes. Thus, this is a task for the research community as a whole, not to be solved in each individual study.

Many of the research approaches mentioned in the typology are not new, and their contributions to quality have been described for generations [129], but their contributions to useful knowledge need more attention. For example, multiple regression and path analysis have long focused on mediators and moderators to contribute to explaining findings. Yet, they have great potential to mitigate the risks and maximize the benefits of EBIs. The risks involved in eliminating a core component can be serious, yet the risk is likely to be low if a core component fails to mediate outcomes in study after study. Likewise, if an implementation strategy is shown to moderate outcomes by increasing the effect sizes in several studies, then end users can safely assume that it is likely to be an important component in new contexts.

Some of these research approaches have a different set of requirements than the established research-to-practice pathway suggests, including a shift in the roles of researchers and end users. This is particularly true for the approaches that turn the tables and consider usefulness upfront, such as when interventions and studies are designed with usefulness in mind. Co-creation and participatory approaches become the guiding words for such approaches. The researchers have expertise in scientific methods and theories but need end users’ expertise on their context and the relevance of outcomes if EBIs are to be useful beyond their own specific study. By working together, it becomes easier to have a dual focus on both usability and scientific quality.

## Conclusions

Researchers need to provide the end users of research findings with relevant information so that EBI can easily be used in practice. The proposed typology presents methodological approaches to be used in intervention research to increase the usefulness of EBIs and thus, invites the research community to consider ways to optimize not only the trustworthiness but also the usefulness of research.

## Data Availability

Not applicable.

## Abbreviations

EBI: Evidence-based interventions
LCGA: Latent class growth analysis
RCT: Randomized controlled trial

## Footnotes

1 Social science describes several kinds of knowledge use. The decisions to adopt and implement an EBI are direct, instrumental uses of evidence. Three other kinds of knowledge use are also important to this process: conceptual use (serious consideration but no direct action), persuasion of others to a course of action, and process use, in which participants’ frame of reference is changed by participating in research or evaluation.
